# Production of reactive oxygen species in macrophages treated with essential oil of *Croton argyrophyllus *Kunth

**DOI:** 10.1186/1753-6561-8-S4-P255

**Published:** 2014-10-01

**Authors:** Silvan Araújo, Aline Oliveira, Matheus Santos, Sandra Santos, Jamile Ferro, Emiliano Barreto, Alexandre Cândido, Brancilene Araujo, Angelo Antoniolli, Charles Estevam

**Affiliations:** 1 Physiology Department, Sergipe Federal University, São Cristóvão, SE, Brazil; 2 Institute of Biological Science and Health, Alagoas Federal University, Maceió, AL, Brazil; 3 Morphology Department, Sergipe Federal University, São Cristóvão, SE, Brazil

## Background

Essential oils are complex systems which consist mainly of volatile compounds of lipophilic source, as terpenes, sesquiterpenes and some non-terpenes [1]. Resulting from secondary metabolism, essential oil from *Croton argyrophyllus *(EOCA) is commonly extracted from its leaves. It is known that the essential oils from Croton species exhibit good antioxidant activity against DPPH free radical and reactive oxygen species (ROS) [2]. Several diseases are attributed to the increase of free radicals that show the importance of endogenous antioxidants like enzyme superoxide dismutase (SOD). Considering the use in folk medicine, it is relevant to find which concentration of EOCA has antioxidant effect against free radicals. To answer this question, the study aimed to evaluate ROS production in peritoneal macrophages of essential oil from *C. argyrophyllus*.

## Methods

After collection in the field, the essential oil from leaves of *C. argyrophyllus *was extracted by hydrodistillation method using apparatus of the type Clevenger having a yeld of 0,5%. Male Swiss mice (n = 5) that were 8 weeks old were used. All procedures were approved by and followed the guidelines of the institutional ethics committee on animal research at Federal University of Sergipe. After being sacrificed, were injected 10 mL PBS, then after vigorous massage was obtained peritoneal exudate. The macrophages were prepared in 96-well plate, 10 µL/well NBT (2.0 mg/mL) and EOCA in different concentrations (5, 10, 25, 50 and 100 µg/mL) [3]. NBT reduction was measured in response to zymosan (250 µg/mL in RPMI). Next, the micro plate was incubated at 37°C for 120 minutes, and the reaction was stopped by discarding the supernatant. The sediment was resuspended by addition of 120 µL DMSO/80 µL KOH (2M) in each well. The results were obtained in micro plate ELISA reader using as a blank the NBT in wells with unstimulated cells. The optical density (OD_630nm_) of formazan produced (insoluble blue deposit) was directly proportional to the ROS generated by phagocyte combined with a lower activity of the enzyme SOD. The data were subjected to variance analysis (ANOVA), with Tukey post hoc. The data variation were tested in triplicate and the differences considered statistically significant when p < 0.05.

## Results and conclusions

Some sesquiterpenes found in the composition of the EOCA have proven antioxidant activity such as bicyclogermacrene [4]. In the NBT test, the EOCA concentrations from 10 µg/mL to 100 µg/mL showed inhibitory activity of SOD and consequently generated more ROS. Thus, the concentration 5 µg/mL was the most effective, both in comparison with the blank and the negative control (Zy). It was concluded that the lower concentrations of 10 µg/mL have antioxidant activities and therefore it is suggested cellular protection against free radicals.
